# Post-Streptococcal Auto-Antibodies Inhibit Protein Disulfide Isomerase and Are Associated with Insulin Resistance

**DOI:** 10.1371/journal.pone.0012875

**Published:** 2010-09-23

**Authors:** Adi Aran, Karin Weiner, Ling Lin, Laurel Ann Finn, Mary Ann Greco, Paul Peppard, Terry Young, Yanay Ofran, Emmanuel Mignot

**Affiliations:** 1 Psychiatry and Behavioral Sciences, Stanford University, Stanford, California, United States of America; 2 Hebrew University, Jerusalem, Israel; 3 Department of Population Health Sciences, University of Wisconsin, Madison, Wisconsin, United States of America; 4 Behavioral Biochemistry Laboratory, SRI International, Menlo Park, California, United States of America; 5 Laboratory of Systems Biology and Functional Genomics, The Goodman Faculty of Life Sciences, Bar-Ilan University, Ramat-Gan, Israel; Albert Einstein Institute for Research and Education, Brazil

## Abstract

Post-streptococcal autoimmunity affects millions worldwide, targeting multiple organs including the heart, brain, and kidneys. To explore the post-streptococcal autoimmunity spectrum, we used western blot analyses, to screen 310 sera from healthy subjects with (33%) and without (67%) markers of recent streptococcal infections [anti-Streptolysin O (ASLO) or anti-DNAse B (ADB)]. A 58 KDa protein, reacting strongly with post-streptococcal sera, was identified as Protein Disulfide Isomerase (PDI), an abundant protein with pleiotropic metabolic, immunologic, and thrombotic effects. Anti-PDI autoantibodies, purified from human sera, targeted similar epitopes in Streptolysin O (SLO, P51-61) and PDI (P328-338). The correlation between post-streptococcal status and anti-human PDI auto-immunity was further confirmed in a total of 2987 samples (13.6% in 530 ASLO positive versus 5.6% in 2457 ASLO negative samples, p<0.0001). Finally, anti-PDI auto-antibodies inhibited PDI-mediated insulin degradation *in vitro* (n = 90, p<0.001), and correlated with higher serum insulin (14.1 iu/ml *vs.* 12.2 iu/ml, n = 1215, p = 0.039) and insulin resistance (Homeostatic Model Assessment (HOMA) 4.1 *vs.* 3.1, n = 1215, p = 0.004), in a population-based cohort. These results identify PDI as a major target of post-streptococcal autoimmunity, and establish a new link between infection, autoimmunity, and metabolic disturbances.

## Introduction

Beta hemolytic streptococcal infections, typically pharyngitis and skin infections, are usually benign, but can lead to serious autoimmune complications such as rheumatic heart disease, Sydenham chorea, and glomerulonephritis [1]. In rheumatic heart disease, probably the most recognized post-streptococcal autoimmune disease, molecular mimicry between streptococcal antigens and selected cardiac proteins, triggers cardiac inflammation, with resulting valvular damage [2], [3]. The full extent of post-streptococcal autoimmunity is still unclear and new post-streptococcal entities have been suggested, notably encephalitis lethargica [4], obsessive-compulsive disorder, and tics [5], [6]. In a recent study, we found a positive correlation between the onset of narcolepsy and the levels of post-streptococcal antibodies [7]. This led us to search for novel post-streptococcal autoantibodies.

Protein Disulfide Isomerase (PDI) is a multifunctional enzyme that primarily catalyzes disulfide bond formation, breakage, and rearrangement [8]. Disulfide bonds stabilize the structure of proteins and can regulate the activity of various enzymes [9] (Fig. S1). PDI is primarily associated with the endoplasmic reticulum (ER) where it participates in protein folding during biosynthesis. It is also found on the cell membrane and may be actively secreted by various cell types [9], [10]. Extracellular PDI has been shown to regulate a number of activities including: cellular adhesion [11], pathogen entry (notably HIV) [12], [13], platelet aggregation and secretion [14], tissue factor pro-coagulant activity (the limiting step in activation of the coagulation cascade) [15], [16], [17], [18], intracellular nitric oxide delivery [19], and insulin degradation [20], [21].

Insulin resistance is rapidly growing in frequency in association with obesity in the developed world [22], and is a core component of metabolic syndrome and diabetes (type 2). Typically, high insulin levels are maintained in the face of high plasma glucose, due to the reduced effects of insulin on fat, liver, and muscle cells. Insulin resistance stimulates triglyceride degradation and increases plasma levels of free fatty acids, while also reducing glucose uptake and glycogen synthesis in the liver and muscle.

Based on the relationship between PDI expression and insulin degradation, we tested the hypothesis that streptococcal infections and the resulting anti-PDI autoantibodies could have deleterious effects on insulin metabolism.

## Results

### PDI is a major target of post-streptococcal autoimmunity

**Figure 1:**
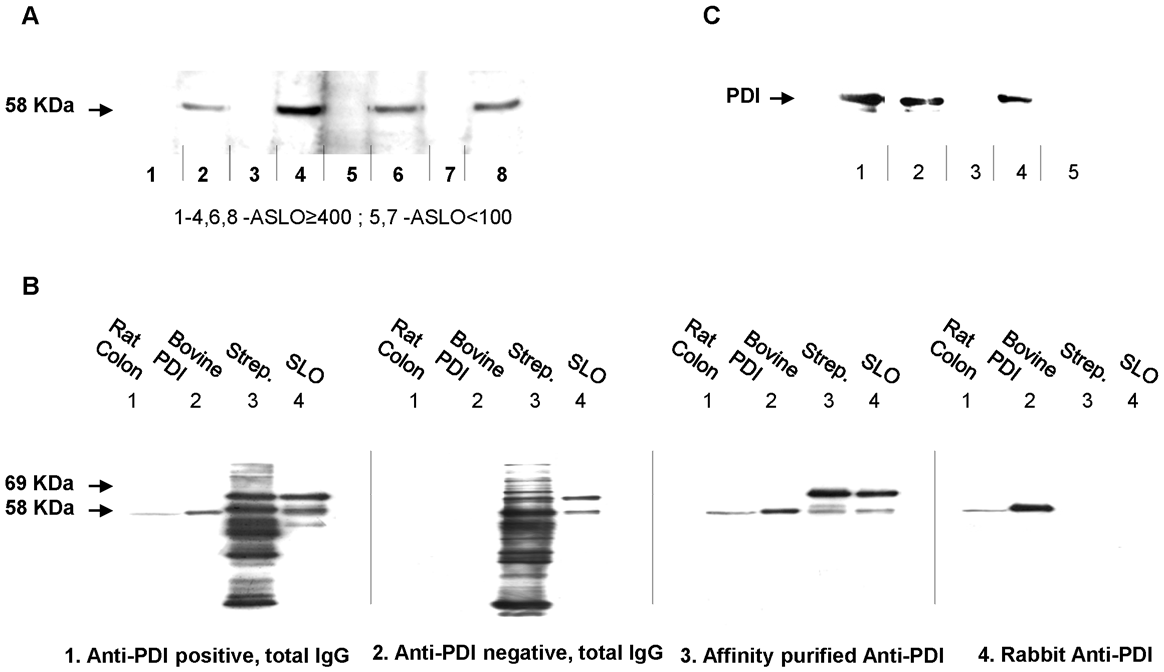
PDI is a target of post-streptococcal autoimmunity. Proteins of various sources (described below) were separated by SDS-PAGE, transferred to membranes, and probed with serum, total IgG, or affinity-purified antibodies extracted from participants. (A) Association of ASLO status with auto-immunoreactivity, as detected by a 58 KDa immunoreactive band. Rat urinary bladder extracts (15 µg/strip) were probed with selected ASLO positive (≥400IU; lanes 1–4, 6 and 8) and negative (<100IU; lanes 5 and 7) sera. In this example 4 of 6 ASLO positive sera are also positive for the 58 KDa band. (B) Reactivity of antibodies with: rat tissue extracts, extracted bovine PDI, streptococcus proteins and purified SLO. Lane 1- rat colon extract (30 µg); lane 2 - purified bovine PDI (100 ng); lane 3 - *Streptococcus pyogenes* – secreted proteins (1.5 µg) and lane 4 – purified Streptolysin O (10 ng). Proteins were immunobloted with: (1) total IgG from subject 2 (Figure 1A); (2) total IgG from anti-PDI negative/ASLO positive subject; (3) affinity purified anti-PDI from subject 6 (Figure 1A) and (4) total IgG from rabbit immuned against bovine PDI -as control. (C) Reactivity of sera and extracted anti-PDI autoantibodies with recombinant human PDI. Recombinant human PDI (1 µg/lane) was immunobloted against total IgG from rabbit immuned against bovine PDI (lane 1); total IgG from subject 6 (lane 2); total IgG from subject 3 (lane 3); affinity purified anti-PDI from subject 6 (lane 4) and affinity purified anti-SLO antibodies (lane 5).

Three hundred and ten healthy participants, (“Stanford exploratory sample”), were screened for the presence of antistreptococcal antibodies, ASLO and ADB. Sixty-five subjects (21%) had ASLO ≥200 IU, 67 (22%) had ADB ≥340 IU and a total of 102 (33%) had ASLO ≥200 IU and/or ADB ≥340 IU (“post-streptococcal sera”). These sera, diluted 1∶250, were next tested for the presence of autoantibodies on western blots of rat colon, liver, intestine, urinary bladder, brain cortex, and brain-stem extracts. We found that post-streptococcal sera reacted ∼6 times more frequently than control sera with a 58 KDa protein (Figure 1A), present in all tested tissues with highest affinity in the colon, liver, and urinary bladder (26.4% versus 4.3% of control sera in colon, n = 310, p<0.0001). Following two-dimensional gel separation, the 58 KDa protein was identified by mass spectrometry (MS) as PDI in liver, colon, and pancreas extracts (data not shown). This was confirmed using purified bovine PDI (Figure 1B- panels 1, 2) and recombinant human PDI (Figure 1C). We next developed an ELISA assay using extracted bovine PDI (1.5 µg/ml bovine PDI, 1∶250 serum dilutions), and used this to test sera from the initial 310 samples, an additional 372 subjects (“Stanford replication sample”), and 1211 participants (2847 sera) of the Wisconsin Sleep Cohort. In all cases, ASLO or ADB positive cases were more likely to be anti-bovine PDI positive (Table 1). The odds ratio (OR) across all cohorts was 4.2 (95%CI: 3.5, 5.2).

**Table 1:** Association between Anti-Streptolysin O (ASLO) and anti-PDI Antibodies.

	*ASLO * *positive$*	*ASLO negative*	*Odds Ratio* *(95% CI)*	*P value*
**Anti-bovine PD** *I* ¶				
Exploratory sample (Stanford)	33.8% (65)	14.7% (245)	3.0 (1.6, 5.5)	<0.0001
Replication (Stanford)	33.3% (99)	11.4% (273)	3.9 (2.2, 6.8)	<0.0001
Wisconsin Sleep Cohort	38.6% (446)	10.2% (2401)	4.6 (3.7, 5.8)	<0.0001
All samples	37.2% (610)	12.2% (2919)	4.2 (3.5, 5.2)	<0.0001
**Anti- human PDI** #				
Exploratory sample (Stanford)	8.3% (24)	7.5% (80)	1.1 (0.2, 5.8)	0.92
Replication (Stanford)	5.9% (85)	3.4% (263)	1.7 (0.6, 5.4)	0.32
Wisconsin Sleep Cohort	15.4% (421)	5.8% (2114)	3.0 (2.1, 4.1)	<0.0001
All samples	13.6% (530)	5.6% (2457)	2.7 (2.0, 3.6)	<0.0001

Results are presented as percentages of positive anti-PDI sera (number of all sera in this subgroup).

$ ASLO positivity defined as ASLO ≥200 for participants ages 6–40 years and ASLO ≥100 for participants older than 40 years, as recommended [40].

¶ Anti-bovine PDI defined as positive if ELISA OD ≥0.5.

# Anti-human PDI defined as positive if ELISA OD ≥0.75.

### Mimicry between PDI and Streptolysin-O (SLO)

**Figure 2:**
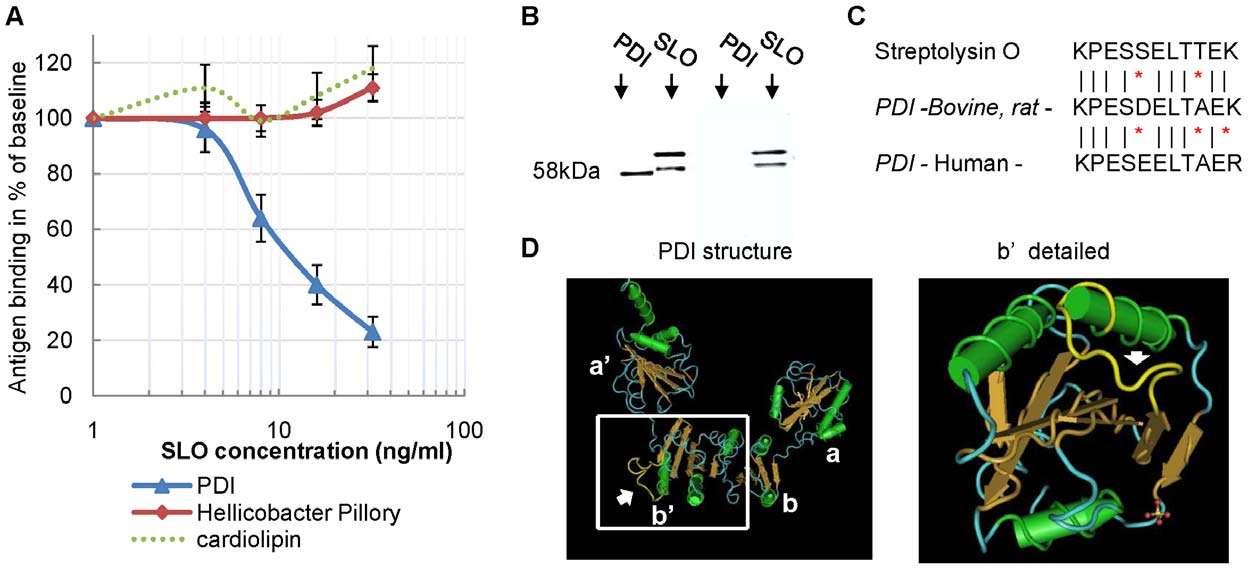
Mimicry between PDI and Streptolysin O (SLO). (A) Competitive ELISA demonstrates a common SLO-PDI determinant. Serum samples (n = 20) positive for anti-PDI, anti-Helicobacter Pillory (HP) and anti-cardiolipin (ACL) antibodies were pre-incubated with increasing amounts of SLO (0–32 ng/µl) and subjected to ELISAs (PDI, HP and ACL). Baselines (100%) were determined by incubation of each serum with PBS only. Results are presented as a mean ± SEM. (B) Reactivity of ASLO antibodies with and without anti-PDI activity. Purified bovine PDI (100 ng) and purified SLO (10 ng) were subjected to SDS gel electrophoresis followed by immunoblotting with Affinity purified anti-SLO antibodies from anti-PDI positive (left) – and anti-PDI negative (right) subjects. (C) Similar immunogenic determinants in SLO and PDI. The 11 amino-acids sequence of the similar determinant is presented in SLO (p51–p61), bovine/rat PDI – (p330-340) and human PDI (p328-338). (D) Location of the similar determinant on the PDI protein (in yellow, pointed by an arrow). Left – structure of all 4 subunits (yeast). Right – focus on the b' subunit (human), an important subunit containing the substrate binding site. Note that the similar determinant is in the exposed area of the binding site. In the SLO protein, the determinant is also exposed (p51-61; not shown).

A bovine PDI affinity column was next used to isolate anti-PDI antibodies from human sera. These purified human autoantibodies were then tested for cross-reactivity with Streptococcal proteins (extracted from *S. pyogenes*). Strong reactivity was observed with a 69 KDa streptococcal protein present in the secreted fraction (Figure 1B- panel 3), a protein later identified as Streptolysin O (SLO) using Mass Spectrometry. Competitive ELISA demonstrated a specific and dose dependent inhibition of PDI binding, after preincubation of anti-PDI positive sera with traces of purified SLO (Figure 2A, ED_50_∼10 ng), indicating that SLO specifically cross-reacts with anti-PDI antibodies.

Importantly however, most ASLO positive sera were not anti-PDI positive. Further, affinity purified anti-Streptolysin O antibodies isolated from ASLO positive/anti-PDI negative sera did not cross-react with PDI (Figure 2B). These results indicate that ASLO antibody testing recognizes several types of antibodies, targeting different SLO epitopes, with only a subset cross-reacting with PDI. Computerized 3-D modeling of SLO and PDI identified an eleven amino acid determinant, similar in SLO (p51-61) and PDI (bovine: p330-340; human: p328-338), and exposed to solvent in both proteins (Figures 2C, 2D). Interestingly, one lysine (K) of the bovine/rat PDI (and SLO), is replaced by an arginine (R) in humans (Figure 2C). We found that this change is associated with a reduced affinity of the antibody for human PDI (as indicated by higher serum concentrations needed to detect binding of the antibody to recombinant human PDI -see methods). Nonetheless, the 11-aminoacid determinant of bovine or human PDI, selectively inhibits binding to PDI in the corresponding species in competitive ELISAs (Fig. S2 A). Further analysis of anti-human PDI antibodies suggest the existence of other determinants in addition to the 11 amino acids region mentioned above (Fig. S2). In addition to the observed association between ASLO and anti-bovine PDI reactivity, strong association between anti-human PDI and ASLO is present (Table 1, see also Table S1). The OR for increased presence of anti-human PDI autoantibodies in conjunction with ASLO across all cohorts was 2.7 (95% CI: 2.0, 3.6).

### Post-streptococcal anti-PDI antibodies inhibit PDI activity

**Figure 3:**
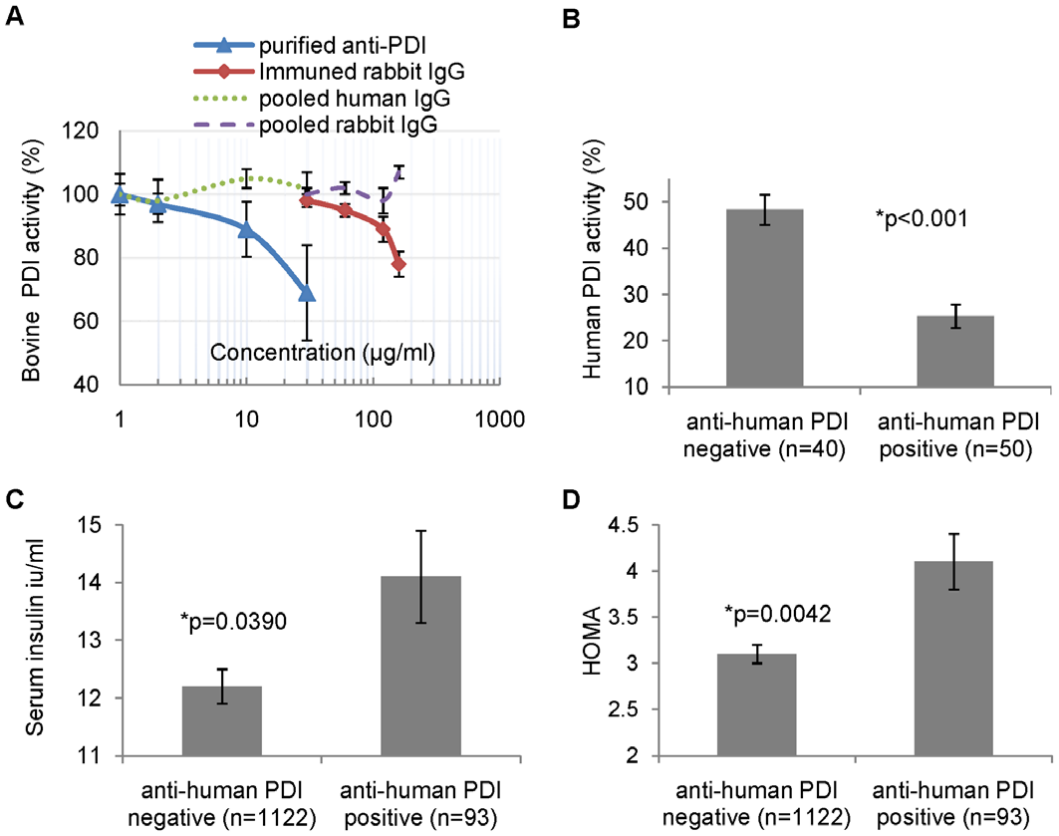
Functional effect of anti-PDI antibodies on PDI activity and association of anti-PDI status with insulin resistance. (**A**) Effects of purified anti-PDI antibodies on purified bovine PDI activity. Purified Bovine PDI activity was measured in the presence of 0–60 µg/ml purified anti- bovine PDI from anti-PDI positive subjects (triangles) and 0–160 µg/ml total IgG from rabbit immuned against bovine PDI (diamonds). Pooled human and rabbit IgG (dashed lines) were used as control. Results are compared to activity in the presence of same volume of PBS (defined as 100%) and presented as a mean ± SEM of three experiments. PDI activity was measured using the insulin transhydrogenase assay. (**B**) Effects of human sera (1∶50) on human recombinant PDI activity. Recombinant human PDI activity was measured in the presence of 20 µl/ml human sera positive or negative for anti-human PDI antibody. Results are presented as mean ± SEM. (**C**) Association of anti- human PDI status with serum insulin levels. Results are presented as mean ± SEM, adjusted for age, gender, education, BMI and smoking. (**D**) Association of anti-human PDI status with insulin resistance. Insulin resistance was estimated using HOMA (Homeostatic Model Assessment of insulin resistance). Results are presented as mean ± SEM, adjusted for age, gender, education, BMI and smoking. For details, see Table S2.

Anti-PDI antibodies, induced by immunization of animals with purified PDI, were shown recently to inhibit PDI function (*in vitro* and *in vivo*) in leukocyte adhesion [11], viral entry [23], platelet aggregation [24], tissue factor activation [16], [17], [18], and insulin degradation [20]. To explore the putative functional effects of the post-streptococcal anti-PDI antibodies identified in this study on PDI activity, we used the insulin turbidity assay. This well-established assay measures the activity of PDI using insulin as a substrate [25]. Both human recombinant PDI and extracted bovine PDI were used. Purified anti-PDI antibodies and diluted sera positive for anti-human and/or anti-bovine PDI strongly inhibited PDI activity (Figure 3 and Fig. S2 B).

### Anti-PDI antibodies are associated with higher serum insulin and insulin resistance

Links between infections, immunity, obesity, and metabolic syndrome were recently described [26], [27]. We hypothesized that the identified anti-PDI antibodies may, at least partially, underlie this association. We tested this hypothesis using samples from the Wisconsin Sleep Cohort, an ongoing longitudinal study of sleep and metabolic indices in Wisconsin state employees [28]. We compared serum insulin and fasting glucose levels in participants with and without anti-human PDI (n = 1215). As hypothesized, participants who were positive for anti-human PDI had significantly higher insulin levels and higher HOMA (Homeostatic Model Assessment of insulin resistance; HOMA  =  insulin x Glucose/405), compared to anti-human PDI negative subjects. The results were adjusted for age, gender, education, BMI, and smoking (Figure 3, Table S2). Sleep Disordered Breathing (Apnea-Hypopnea Index) had no effect on the association.

In samples of the same participant tested 4 years apart, the anti PDI positivity for successive visits was correlated (Kappa = 0.32, 95% CI: 0.22, 0.42, p<0.0001, n = 1118 subjects with ≥2 visits), with 33% of samples positive at baseline remaining positive 4 years later, suggesting moderately long stability for this autoantibody (years but not decades).

## Discussion

SLO is a highly immunogenic streptococcal toxin that induces ASLO antibodies in a majority of subjects following streptococcal pharyngitis, hence its clinical utility as a marker. Titers typically increase after 2 weeks, peak at 2–4 months, and decrease thereafter [29]. In most cases, these antibodies do not target the SLO-p51-61 epitope, which we identified to be homologous to PDI- p328-338, an epitope located within the PDI binding site and crucial to its function. In about 8% of the general population, however, molecular mimicry with human PDI occurs (Table 1), with functional effects on the enzyme activity (Figure 3). Importantly, the human serum dilution used in the enzyme inhibition studies (1∶50, similar to that used in the ELISA assays) is compatible with physiological effects *in vivo*.

The higher affinity of the human anti-SLO-p51-61 antibodies to the bovine/rat antigens (which share higher homology to SLO than does the human antigen), made the isolation processes possible, and was a bridge to the identification of the anti-human PDI.

**Figure 4:**
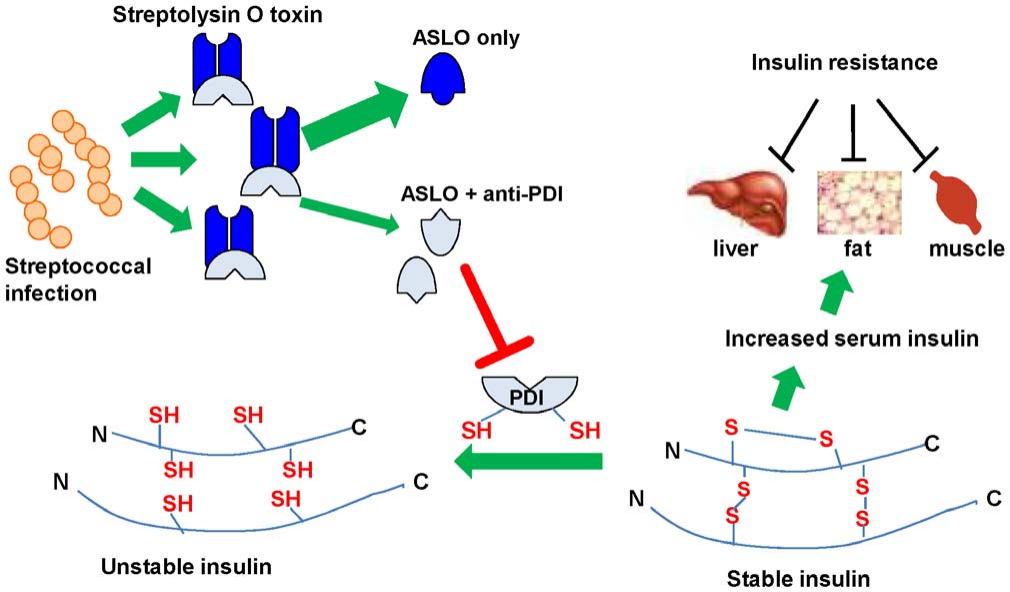
Possible pathway mediating insulin resistance in subjects with post-streptococcal anti-PDI autoantibodies. Streptococcal infections induce production of anti-Streptolysin O antibodies (ASLO), some of which share a common determinant with PDI. Molecular mimicry leads to inhibition of PDI which among other functions reduces and degrades insulin. The disulfide bonds of insulin are generated on the preproinsulin peptide, and thus reduction of any of the two disulfide bonds of insulin results in irreversible changes in activity. We hypothesize that lower degradation of insulin by PDI in the presence of anti-PDI antibodies result in higher insulin levels and insulin resistance.

Here we suggest, for the first time, that beta hemolytic streptococcal infections may contribute in some cases to insulin resistance. Although the presence of an autoantibody does not necessarily mean an autoimmune disease, the functional characteristics of anti-PDI combined with the epidemiological associations with altered insulin metabolism suggest an autoimmune contribution to the pathogenesis of insulin resistance. A possible pathway is reduced insulin degradation by the anti-PDI, that leads to higher insulin and eventually insulin resistance [30] (Figure 4).

We used the homeostatic model assessment (HOMA) to assess insulin resistance. While HOMA has been validated against a variety of physiological methods and its use in cohort and epidemiological studies is considered appropriate, it is not a definitive measurement of insulin resistance and should be interpreted cautiously [31]. In our study an average HOMA of 3.1 was found in participants without anti-PDI antibodies compared to 4.1 (33% higher) in participants positive for this autoantibody. As cutoff of 3.6 to 4.6 has been suggested for the diagnosis of insulin resistance (depends on the BMI of the tested individual) [32], this HOMA difference between the groups is probably of clinical significance. Nevertheless, the use of definitive techniques for the measurement of insulin resistance should be considered in future studies including the euglycemic insulin clamp technique, the glucose tolerance test and the insulin suppression test.

Multiple genetic and environmental factors are probably involved in the pathogenesis of insulin resistance. This study suggests that one of these genetic-environmental combinations is the tendency to produce and maintain anti-PDI antibodies following Streptococcal infection. Further studies are needed to explore the associations between anti-PDI antibodies, HLA (and other genetic markers using genome wide association), family history of type 2 diabetes, and Insulin resistance.

As PDI is a pleiotropic enzyme, additional effects of this functional autoantibody are likely. Most notably, extracellular PDI is now recognized as a key player in platelet activation and fibrin formation [14], [15], [16], [17], [18]. *In-vitro* and *in-vivo* studies have shown functional effects of experimentally induced anti-PDI antibodies on platelet activation mediated by the platelet Fc receptor [33], and fibrin formation[17]. Extracellular PDI is also involved in resistance to infection [12], [13], cardiovascular disease [19], [34], and immune recognition of cancer [35], [36], [37]. Our findings broaden the spectrum of post-streptococcal immunity and suggest effects on insulin metabolism. Further clinical studies, encompassing also other areas of medicine where PDI has a role, are clearly needed to determine the full clinical impact of this novel autoantibody.

## Methods

### Ethics Statement

The Study was approved by the institutional review boards of Stanford and Wisconsin Universities, and a written informed consent was obtained from all participants. Animal studies were carried out in strict accordance with the recommendations in the Guide for the Care and Use of Laboratory Animals of the National Institutes of Health. The protocol was approved by the Institutional Animal Care and Use Committee (IACUC)of Stanford University (Permit Number:13985).

### Subjects

Stanford cohorts included 310 and 372 healthy volunteers (mean age 25.9±11.8 years (range 3.4–86 years), 58% females). The Wisconsin Sleep Cohort [28] is an ongoing longitudinal study. Metabolic and sleep data, and blood samples are obtained from each participant every four years (2,847 visits/serum samples in 1211 participants). Mean age at blood draw 55.5±8.6 years (range 33.1–74.5); 45.5% females.

### Antibodies, Reagents, and Commercially available Assays

Proteins and Antigens: bovine PDI, Streptolysin O, and bovine insulin were obtained from Sigma-Aldrich; PDI recombinant human protein - from Assay Designs; Short peptides specifically designed for this study - from GenScript. Antibodies: Rabbit polyclonal anti-bovine PDI, human IgG, and rabbit IgG from Sigma-Aldrich; Donkey anti-human IgG conjugated with horseradish peroxidase - from Jackson ImmunoResearch Laboratories. Assays: Insulin - from Linco Research; ASLO – SeraTest ASO, Remel; ADB - Streptonase-B, Wampole Laboratories; Anti-Helicobacter Pylori IgG ELISA - H. pylori IgG BioCheck; Anti-cardiolipin IgG ELISA - Cardiolipin IgG ELISA, Calbiotech. Other kits: Reversible protein detection kit - Sigma-Aldrich; Montage Antibody Purification Kit with PROSEP-G media- Millipore; Chemiluminescence ECL kit - Thermo scientific; Tetramethylbenzidine (TMB) kit- Vector Laboratories.

### Western Analyses

Tissue collected from Wistar rats were homogenized in RIPA buffer, and centrifuged (17,000× g; 20 min, 10°C). The supernatant was denatured (85°C, 5 min), electrophoresed (10% acrylamide gel), and transferred to 0.45-µm nitrocellulose (100V, 80 min). Fifteen to 30 µg tissue extract (or 1 µg purified protein) were loaded in each lane. Membranes were stained reversibly stained (kit) to confirm homogeneity of protein loading, and cut in separate strips. Strips were blocked (5% milk in PBS; 90 minutes), and incubated with individual human sera (diluted 1∶250–500) or total IgG (purified from serum using Montage Antibody Purification Kit; 10 µ/ml; 120 minutes, room temp), and then with secondary antibody (Donkey anti-human IgG -HRP, 1∶25,000). Immunoreactive proteins were visualized using ECL kit according to the manufacturer's instructions.

### Isoelectric focusing/SDS-PAGE two-dimensional electrophoresis (2-DE)

Isoelectric focusing (IEF) buffer was prepared with 2% (volume/volume) ampholyte (pH 3.7–9.2). Rat tissue extracts (2.5 mg, prepared in IEF lysis buffer) were dissolved in a final IEF buffer volume of 2.5 ml, applied to MicroRotofor (Bio-Rad), and electrophoresed for 1.5–2.5 hours (1W constant power, 4°C). Protein fractions (10×200 ìl) were harvested, and submitted to SDS-PAGE (12% or 15% polyacrylamide).

### Extraction of Streptococcus pyogenes antigens

*S. pyogenes* was grown in Todd-Hewitt broth (18 hrs, 37°C) without agitation. Extracellular (CS) antigens were obtained by precipitation of culture supernatant (1500 ml) with 80% saturated (NH_4_)_2_SO_4_. After centrifugation (11000×g, 60 min, 10°C), proteins were suspended in 6 ml PBS or IEF buffer, dialyzed (4°C overnight against dH_2_O), and filter sterilized.

### Mass Spectrometry identification of immunoreactive bands

Following IEF and SDS–PAGE, gels were stained (GelCode Blue, PIERCE), target bands excised, and subjected to tryptic digestion according to the instructions of the UCSF Mass Spectrometry Core facility (http://donatello.ucsf.edu/ingel.html). Tryptic fragments were analyzed by mass spectrometry (MALDI-TOF system,Voyager DE-PRO, Applied Biosystems, BMSF, UNSW). MOWSE probability score, sequence coverage, pI, and molecular weight values were used to identify the most likely candidate protein for each band.

### Anti-PDI determination using ELISA

Microtiter plates (96 wells) were coated with bovine (100 µl of 1.5 µg/mL) or recombinant human (100 µl of 5 µg/mL) PDI (incubated at 4°C, 24 hours). Plates were rinsed and flicked 3x, blocked (5% milk), and exposed to human diluted sera (90 min, room temperature). Anti-bovine PDI antibodies were detected using a 1∶250 dilution; anti-human PDI antibodies using a 1∶50 dilution. Donkey anti-human IgG-HRP (1∶10,000 ) was used as the secondary antibody. To detect antibody–antigen interactions tetramethylbenzidine (TMB) was added (reaction stopped at 5 min with 50 µl H3PO4 (1M)), and the resulting signal read at 450 nm (Molecular Devices EMAX micro plate reader). The cutoff for positive was OD ≥0.5 for anti-bovine PDI and OD≥0.75 for anti-human PDI (due to higher background). All samples were assayed in duplicate.

### Affinity purification of anti-PDI and ASLO Antibodies

Total IgG from anti-bovine PDI positive and/or ASLO positive sera were affinity-purified using bovine PDI (Affinity Life Sciences, Inc) or SLO (AminoLink Plus Immobilization Kit, Thermo Scientific).

### Insulin transhydrogenase assays

Bovine insulin (250 µg) was suspended in 250 µl of TRIS 50 mM, EDTA 2 mM, PH-7.5. Bovine or recombinant human PDI (0.5 µg) was added, and reaction initiated with 1 µl of 0.1M DTT (dithiothreitol). The transhydrognation of insulin was monitored by absorbance (OD_650nm_) over a 70 minute period. Inhibitors of PDI -mediated insulin transhydrogenation were pre-incubated with PDI and insulin for 45 minutes prior to the addition of DTT.

### Epitope modeling

Several models for the structure of SLO and the yet unresolved fragments of PDI were generated using modeller [38] and I-TASSER [39] These models were manually refined prior to analysis for a common structural determinant.

### Statistical analysis

Unless otherwise specified, data is presented as mean ± SD. Group comparisons were primarily made using Pearson's χ^2^ or Student's t-tests. In selected cases, multivariate analyses were used to control for possible covariates of interest. Finally, in studies of the Wisconsin cohort, glucose metabolic variables were compared on repeated observations using weighted average, with control of known confounders (Table S2). The statistical packages SYSTAT or SAS were used for these analyses.

## Supporting Information

Figure S1 PDI regulates polypeptides structure and function. (A) The reduced amino acid cysteine has a sulphur atom (S), as a thiol group (SH). (B) Disulfide bonds (bridge) are covalent bonds formed between two sulfur atoms across two cysteine residues on a protein or polypeptide, which stabilizes the protein/polypeptide tertiary structure. (C) Oxidized PDI binds proteins with thiol groups (C1) and uses its own disulfide bond to oxidize thiols on the target protein, forming a disulfide bridge (C2). The resulting structural change can activate/deactivate target proteins. Reduced PDI binds proteins with disulfide bridges (C2) and can either reduce them to thiols (C1) or change the disulfide bridges (C3), thus altering the protein structure.
(1.34 MB TIF)

Figure S2 Heterogeneity of epitopes in anti-human PDI autoantibodies, and impact on PDI inhibition. (A) Serum samples, positive for anti-bovine PDI, anti-human PDI, or both, were pre-incubated with: 1) PBS; 2) synthetic peptide of the human PDI 11 amino acids determinant -p328-338 (Figure 2C, 100 ng/µl); 3) synthetic peptide of the bovine PDI 11 amino acids determinant-p330-340 (Figure 2C, 100 ng/µl). Treated sera were subjected to anti-bovine PDI ELISA (left) and to anti-human PDI ELISA (right). Results are presented as mean ±SEM of 3 experiments. Rabbit induced anti-PDI was used as positive control. The results suggest at least two types of anti-human PDI determinants. One targets the 11aa determinant similar in PDI and SLO (targets both human and bovine PDI and inhibited by both determinants). The other type(s) (human-PDI specific only) target a different determinant on the human-PDI protein and are not inhibited by the PDI-SLO 11 aa similar determinant (as the rabbit serum anti-PDI control). (B) Impact of anti-human PDI antibodies on recombinant human PDI activity, as measured by the insulin transhydrogenase assay (see Figure 3). The activity was measured when PBS was added to the reaction mix (defined as 100% activity) versus sera (20 µl/ml ) was added. Sera positive for both anti human and anti bovine PDI (right bar) are contrasted with sera positive for anti-human PDI only (middle) and with sera negative for anti-human PDI and anti-bovine PDI (left). Results are presented as mean ± SEM. Human PDI inhibition by sera positive for both antibodies was stronger compared to inhibition by sera positive for anti-human PDI only (P<0.001).
(0.58 MB TIF)

Table S1 Characteristics of participants positive for anti-human and bovine PDI antibodies. Data is presented as number (%) of studies or as mean (SEM) for age. #: Adjusted with ASO status; * Caucasians vs. all other ethnic groups.
(0.06 MB PDF)

Table S2 Serum insulin, glucose and HOMA by anti-human PDI status. Data is presented as mean (SEM) or as N-number of studies (%). * Adjusted for age, BMI, gender, smoking and education. HOMA - Homeostatic Model Assessment of insulin resistance. Note: A higher value indicates increased resistance.
(0.04 MB PDF)
