# Supplementary figures and images for: Post-Streptococcal Auto-Antibodies Inhibit Protein Disulfide Isomerase and Are Associated with Insulin Resistance
Source: PLoS One. 2010 Sep 23;5(9):e12875. doi: 10.1371/journal.pone.0012875 (PMC2944800; doi:10.1371/journal.pone.0012875)

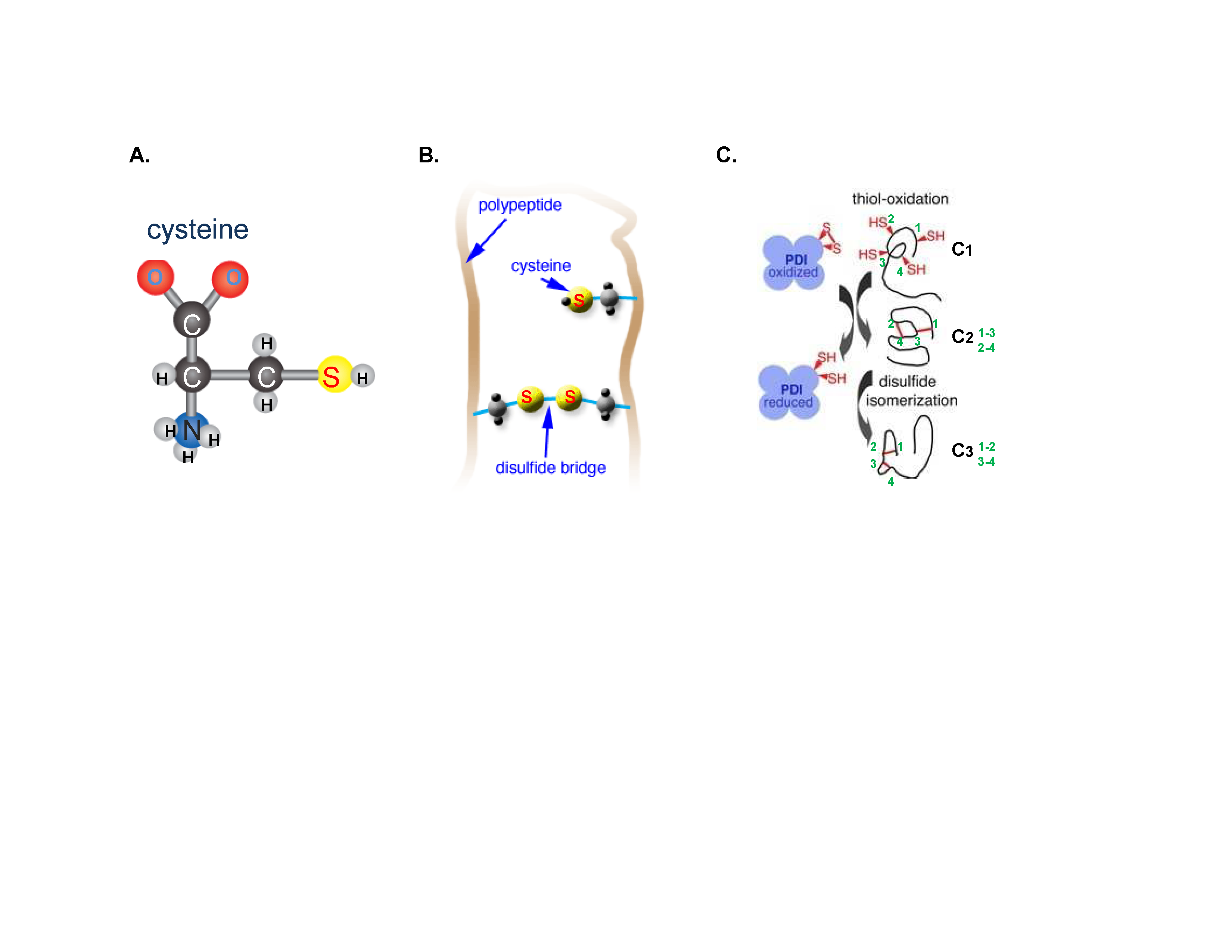

Supplement: Figure S1 — PDI regulates polypeptides structure and function. (A) The reduced amino acid cysteine has a sulphur atom (S), as a thiol group (SH). (B) Disulfide bonds (bridge) are covalent bonds formed between two sulfur atoms across two cysteine residues on a protein or polypeptide, which stabilizes the protein/polypeptide tertiary structure. (C) Oxidized PDI binds proteins with thiol groups (C1) and uses its own disulfide bond to oxidize thiols on the target protein, forming a disulfide bridge (C2). The resulting structural change can activate/deactivate target proteins. Reduced PDI binds proteins with disulfide bridges (C2) and can either reduce them to thiols (C1) or change the disulfide bridges (C3), thus altering the protein structure. (1.34 MB TIF) [file pone.0012875.s001.tif]

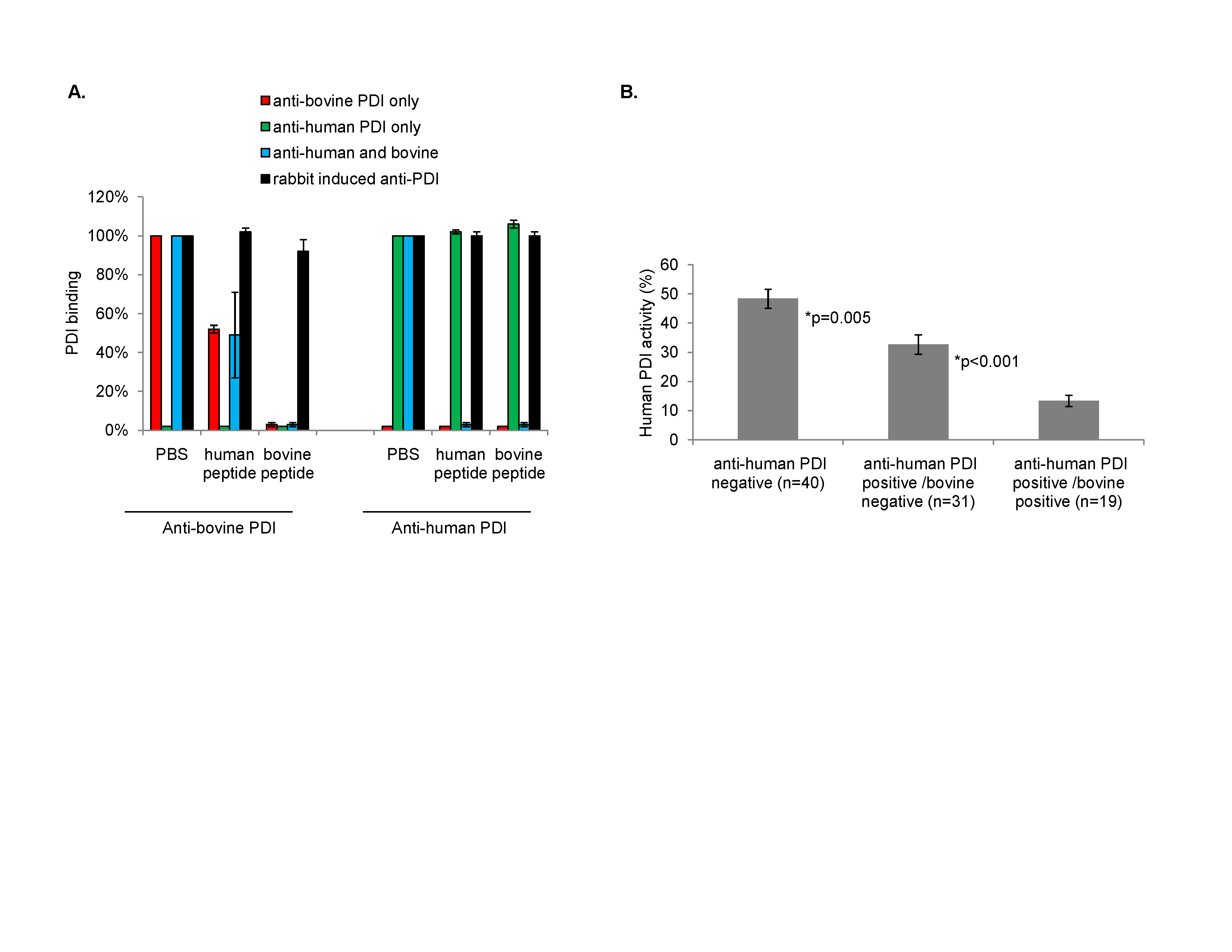

Supplement: Figure S2 — Heterogeneity of epitopes in anti-human PDI autoantibodies, and impact on PDI inhibition. (A) Serum samples, positive for anti-bovine PDI, anti-human PDI, or both, were pre-incubated with: 1) PBS; 2) synthetic peptide of the human PDI 11 amino acids determinant -p328-338 (Figure 2C, 100 ng/µl); 3) synthetic peptide of the bovine PDI 11 amino acids determinant-p330-340 (Figure 2C, 100 ng/µl). Treated sera were subjected to anti-bovine PDI ELISA (left) and to anti-human PDI ELISA (right). Results are presented as mean ±SEM of 3 experiments. Rabbit induced anti-PDI was used as positive control. The results suggest at least two types of anti-human PDI determinants. One targets the 11aa determinant similar in PDI and SLO (targets both human and bovine PDI and inhibited by both determinants). The other type(s) (human-PDI specific only) target a different determinant on the human-PDI protein and are not inhibited by the PDI-SLO 11 aa similar determinant (as the rabbit serum anti-PDI control). (B) Impact of anti-human PDI antibodies on recombinant human PDI activity, as measured by the insulin transhydrogenase assay (see Figure 3). The activity was measured when PBS was added to the reaction mix (defined as 100% activity) versus sera (20 µl/ml ) was added. Sera positive for both anti human and anti bovine PDI (right bar) are contrasted with sera positive for anti-human PDI only (middle) and with sera negative for anti-human PDI and anti-bovine PDI (left). Results are presented as mean ± SEM. Human PDI inhibition by sera positive for both antibodies was stronger compared to inhibition by sera positive for anti-human PDI only (P<0.001). (0.58 MB TIF) [file pone.0012875.s002.tif]
