# Supplementary material for: Post-Streptococcal Auto-Antibodies Inhibit Protein Disulfide Isomerase and Are Associated with Insulin Resistance
Source: PLoS One. 2010 Sep 23;5(9):e12875. doi: 10.1371/journal.pone.0012875 (PMC2944800; doi:10.1371/journal.pone.0012875)
Supplement: Table S1 — Characteristics of participants positive for anti-human and bovine PDI antibodies. Data is presented as number (%) of studies or as mean (SEM) for age. #: Adjusted with ASO status; * Caucasians vs. all other ethnic groups. (0.06 MB PDF) [file pone.0012875.s003.pdf]

	Anti-human PDI				Anti-bovine PDI			
	negative	*Positive*	*p-value*	*Adjusted p-value #*	*negative*	*positive*	*p-value*	*Adjusted p-value #*
Stanford cohorts	N=430 (95%)	N=22 (5%)			N=558 (82%)	N=124 (18%)		
Age, mean (SE)	24.6 (0.3)	27.2 (2.2)	0.044	0.023	26.0 (0.5)	25.5 (1.1)	0.650	0.598
Male Sex, N (%)	169 (39%)	5 (23%)	0.133	0.160	227 (40.6%)	61 (49.2%)	0.083	0.030
Ethnicity, N (%)								
Caucasian	269 (63%)	11 (50%)	0.276	0.211	383 (69%)	109 (88%)	<0.001	<0.001
African-American	55 (13%)	6 (27%)	0.034	0.030	59 (11%)	4 (3%)	0.011	0.017
Asian	104 (24%)	5 (23%)	0.915	0.981	103 (18%)	11 (9%)	0.010	0.026
Wisconsin cohort	N=2289 (92%)	N=182 (8%)			N=2087 (84%)	N=384 (16%)		
Age, mean (SE)	55.2 (0.2)	54.4 (0.4)	0.034	0.208	55.4 (0.2)	53.3(0.4)	<0.001	<0.001
Male Sex, N (%)	1236 (54%)	106 (58%)	0.537	0.433	1113 (53%)	229 (60%)	0.044	0.036
Ethnicity, N (%)								
Caucasian	2220 (97%)	173(97.3%)	0.960*	0.710*	2022 (96.9%)	375 (97.7%)	0.480*	0.788*
African-American	19 (0.8%)	2 (1.1%)			20 (1%)	1 (0.3%)		
Asian	25 (1.1%)	1 (0.6%)			21 (1%)	5 (1.3%)		
At least some college, N (%)	1718 (75%)	143 (79%)	0.554	0.519	1553(74%)	308 (80%)	0.136	0.112
