# Supplementary material for: Post-Streptococcal Auto-Antibodies Inhibit Protein Disulfide Isomerase and Are Associated with Insulin Resistance
Source: PLoS One. 2010 Sep 23;5(9):e12875. doi: 10.1371/journal.pone.0012875 (PMC2944800; doi:10.1371/journal.pone.0012875)
Supplement: Table S2 — Serum insulin, glucose and HOMA by anti-human PDI status. Data is presented as mean (SEM) or as N-number of studies (%). * Adjusted for age, BMI, gender, smoking and education. HOMA - Homeostatic Model Assessment of insulin resistance. Note: A higher value indicates increased resistance. (0.04 MB PDF) [file pone.0012875.s004.pdf]

	Anti-human PDI		
	**Negative**	**Positive**	**p-value**
	N=2289	N=182	
**Age, mean (SE)**	55.2 (0.2)	54.4 (0.4)	0.034
**BMI, mean (SE)**	31.4 (0.2)	31.4 (0.3)	0.876
**Male Sex, N (%)**	1236 (54%)	106 (58%)	0.537
**Smoking, N (%)**			
**Never**	1116 (49%)	95 (52%)	Ref.
**Past**	886 (39%)	67 (37%)	0.804
**Current**	287 (13%)	20 (11%)	0.506
**At least some college, (%)**	1718 (75%)	143 (79%)	0.554
			**Adjusted p-value***
**Glucose (SE) ***	103 (0.8)	103 (2.0)	0.8470
	N=1122	N=93	
**Insulin (SE) ***	12.2 (0.3)	14.1 (0.8)	0.0390
**HOMA (SE) ***	3.1 (0.1)	4.1 (0.3)	0.0042
